# Optimization of the autoclave preparation process for improving resistant starch content in rice grains

**DOI:** 10.1002/fsn3.1528

**Published:** 2020-03-20

**Authors:** Yunzhan Zheng, Zhencheng Wei, Ruifen Zhang, Yuanyuan Deng, Xiaojun Tang, Yan Zhang, Guang Liu, Lei Liu, Jiajia Wang, Na Liao, Mingwei Zhang

**Affiliations:** ^1^ College of Life Sciences Yangtze University Jingzhou China; ^2^ Sericultural & Agri‐Food Research Institute, Guangdong Academy of Agricultural Sciences Key Laboratory of Functional Foods, Ministry of Agriculture and Rural Affairs Key Laboratory of Agricultural Products Processing Guangzhou China

**Keywords:** glycemic index, process optimization, resistant starch, rice grains

## Abstract

The autoclave preparation process to increase the content of resistant starch (RS) in rice grains was optimized, and the results showed that the optimal preparation process was obtained with a water content of 41.63%, a pH of 5.95, an autoclave time of 60.96 min, and a refrigeration time of 17.11 hr. Under these conditions, the theoretical value of RS content in rice grains reached 17.57%. After autoclaving, the estimated glycemic index (EGI) in rice grains was reduced from 78.35 to 66.08 measured after cooking, suggesting that autoclaving was capable of increasing the RS content in rice grains and reducing its EGI value. These results may help spark new concepts and methods for the development of specialized foods for specific populations, such as people with diabetes.

## Graphical abstract

The autoclave preparation process to increase the content of resistant starch (RS) in rice grains was optimized. The RS content of autoclaved rice was increased and the estimated glycemic index (EGI) decreased.

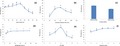

## 1 INTRODUCTION

The *Report on Nutrition and Chronic Diseases of Chinese Residents (2015)* issued by China National Health and Family Planning Commission shows that the prevalence of diabetes in China reached 9.7% in 2012, and the number of patients affected reached 92.4 million. According to the report from the International Diabetes Federation (2017), there were 425 million patients with diabetes worldwide in 2017, while the number of patients with diabetes in China reached 114 million in the same year.

Food intake has a big impact on blood sugar level of diabetic patients. Ingestion of foods containing high levels of resistant starch (RS) can reduce blood sugar levels after meals and promote insulin secretion (Björck et al., 1987; Fuentes‐Zaragoza, Riquelme‐Navarrete, Sánchez‐Zapata, & Pérez‐Álvarez, 2010), as well as improve lipid composition (Fuentes‐Zaragoza et al., 2010). RS has many other physiological functions such as prebiotic effect, hypocholesterolemic effect, and prevention of colonic cancer. (Fuentes‐Zaragoza et al., 2010; Fuentes‐Zaragoza et al., 2011; Haralampu, 2000; Nugent, 2010; Sajilata, Singhal, & Kulkarni, 2006). RS cannot be digested in the small intestine of humans, but can be fermented or partially fermented in the large intestine. RS cannot be hydrolyzed by a mixture of several enzymes such as amylases, glucoamylase, and invertase after 120 min of hydrolysis in in vitro experiments (Alexander, 1995; Englyst, Kingman, & Cummings, 1992). Foods with a low glycemic index (GI) have the characteristics of slow absorption and sustained release of energy, which help maintain blood glucose homeostasis, thereby preventing the incidence of various chronic diseases and improving the health status of the patients.

However, staple foods such as rice have a high GI value (Kumar et al., 2018; Meera, Smita, Haripriya, & Sen, 2019). Rice is the main staple food for more than half of the world's population, especially for people in Asia (Osman, Mohd‐Yusof, & Ismail, 2017). However, rice, featuring a low RS content and a high GI value, is not considered an ideal staple food for diabetics (Kumar et al., 2018; Meera et al., 2019). Kumar et al. (2018) reported the RS contents of 24 rice genotypes between 0.35% and 2.57%, and GI values between 60.07 and 70.36, and a significant negative correlation existed between GI and RS. Therefore, it is important to improve the RS content in rice and reduce the GI of rice to make it suitable for diabetics, so that the nutritional status of diabetics can be improved and the occurrence of diabetic complications can be delayed or even avoided.

It is well known that resistant starch can be classified into five types: physically embedded starch (RS_1_), RS granules (RS_2_), retrograded starch (RS_3_), chemically modified starch (RS_4_), and amylose–lipid complexed starch (RS_5_) (Hasjim et al., 2010; Masatcioglu, Sumer, & Koksel, 2017; Okumusa, Tacer‐Caba, Kahraman, & Nilufer‐Erdil, 2018). Among them, RS_3_ stands out due to its stable physical and chemical properties, and it is currently a popular field of starch research (Dupuis, Liu, & Yada, 2014). Currently, the conventional preparation methods of RS_3_ mainly include the autoclave method (Hu & Zhang, 2010; Li, Fang, & Huang, 2010), enzymatic method (Hu & Zhang, 2010; Wu et al., 2010), extrusion (Masatcioglu et al., 2017), and microwave conversion (Mutlu, Kahraman, & Öztürk, 2017), as well as heating and cooling (Arcila & Rose, 2015). Li et al. (2010) autoclaved rice starch to obtain a RS content of 9.54%. Hu and Zhang (2010) prepared corn RS by an autoclave‐enzymatic hydrolysis method with a yield of 26.34%. Wu et al. (2010) used broken rice starch as a raw material to prepare RS by a microwave‐assisted enzymatic method with a yield of 21.81%.

Among the preparation methods, the autoclave method has the highest potential for commercialization (Zhao, Yu, Liu, Zhou, & Cao, 2013), while other methods are not suitable for industrial application due to high cost, low efficiency, complicated operation steps, and specialized equipment involved, etc. (Hao, Zhang, Guo, Zang, & Yu, 2014; Lan et al., 2015; Masatcioglu et al., 2017; Zhou, Zhang, Meng, Wang, & Yuan, 2014). At present, most of the researches (Ashwar et al., 2016; Björck et al., 1987; Hu & Zhang, 2010; Ji, Yu, Wang, Lin, & Wang, 2010; Li, Zheng, Zheng, & Zheng, 2015; Li et al., 2010; Li, Sun, Wang, & Zhu, 2009; Liu, Yang, Wang, & Tang, 2011; Pan et al., 2008; Song, Zhang, An, Cai, & Peng, 2014; Yang, Pan, Liu, & Tang, 2010; Zhao et al., 2013) around the world focus on autoclave preparation and on how to increase the content of RS by optimizing processing technology from prepared starch. The disadvantages of such processes using starch as starting material are as follows: (a) It wastes a large amount of protein and other nutrients, as well as active ingredients present in agricultural products by extracting starch from such products; (b) it is difficult to dry the starch due to gelatinization after the starch slurry is heated and refrigerated; and (c) it is also prone to deterioration during the drying process. In addition to starch, rice contains protein, minerals, vitamins, and other nutrients that are beneficial to human health, as well as active ingredients such as oryzanol. Therefore, intact rice grains were used as the raw material in this study to improve the RS content of rice by optimizing the RS_3_ preparation conditions, thereby reducing the rice GI value. Results from this study may contribute toward making rice a future staple food suitable for diabetics or a specialized food ingredient for people with special needs.

## 2 MATERIALS AND METHODS

### 2.1 Materials

The rice cultivar “Hefengyouzhan,” which was developed by the Rice Research Institute of Guangdong Academy of Agricultural Sciences, and harvested in the autumn of 2017, was selected. It is a regular *indica* (*Oryza sativa* L.) rice cultivar. A mass of 100 g of rice contained 72.4 g of carbohydrates (subtraction method), 9.2 g of protein (Kjeldahl method), 13.2 g of moisture (gravimetric method), and 1.1 g of ash (calcination method). The proportion of RS in the total starch before and after cooking was 80.1% and 1.9%, respectively.

### 2.2 Reagents and instruments

Porcine pancreatic α‐amylase and glucoamylase (amyloglucosidase from aspergillus niger, CAS number 9032‐08‐0, enzyme activity 100,000 U/g) were purchased from Shanghai Yuanye Biotechnology. 3,5‐Dinitrosalicylic acid, sodium potassium tartrate, and sodium sulfite were purchased from the Damao Chemical Reagent Factory. Crystalline phenol, sodium hydroxide, and anhydrous sodium acetate were purchased from Tianjin Fuchen Chemical Reagent Factory. Glacial acetic acid was purchased from Tianjin Kermel Chemical Reagent, and glucose was purchased from Guangzhou Chemical Reagent Factory. The reagents used were all of analytical grade.

The constant temperature oscillator was purchased from Changzhou Aohua Instrument, the QUINTIX224‐1CN electronic balance was obtained from Sartorius Scientific Instruments, and the UV‐1800 UV–Visible Spectrophotometer was purchased from Shimadzu Corp.

### 2.3 Experimental design

#### 2.3.1 Single factor test

When testing the effect of a single factor on RS content, the following general treatment procedure was adopted: Rice (intact white rice grains, 20 g) was weighed and transferred to a lidded aluminum box. Distilled water was added to the aluminum box until the percentage of water in the mixture reached 50% (weight ratio of the distilled water to the mixture), the pH was then adjusted to 6.0, and the sample was immediately placed in the autoclave and treated at 121°C for 60 min, after which the sample was taken out, naturally cooled for 30 min, placed in a refrigerator at 4°C for 12 hr, and oven‐dried at 60°C for 18 hr for later use.

The added water content (the percentage of distilled water in the mixture at 0, 10%, 20%, 30%, 40%, 50%, 60%, and 70%), autoclave treatment time (20, 40, 60, 80, and 100 min), cooling methods (natural cooling, and rapid cooling at 4°C), refrigeration time (3, 6, 12, 24, 36, and 48 hr), pH value (4.0, 5.0, 6.0, 7.0, and 8.0), and number of treatment cycles (1, 2, 3, 4, and 5) were tested to determine the effect of these factors on RS content. When conducting the experiments on the effect of a single factor, the various conditions for that factor mentioned above were used, while the other conditions in the general treatment procedure were not changed.

#### 2.3.2 Optimization by response surface test

According to the results of the single factor test, four factors, that is, water content, autoclave time, pH value, and refrigeration time, were selected. The four‐factor three‐level response surface test was designed according to the Box–Behnken design principle in the Design‐Expert 8.0.5 software.

### 2.4 Procedures for determination and analysis

#### 2.4.1 Plotting of a standard curve

Analytically pure glucose (100 mg), which was prebaked to a constant weight, was accurately weighed, placed in a beaker, dissolved in a small amount of distilled water, and transferred to a 100‐mL volumetric flask. The solution was diluted with distilled water, shaken, and refrigerated. The 3,5‐dinitrosalicylic acid (DNS, 6.3 g) and 262 ml of NaOH solution (2 mol/L) were added to 500 ml of a hot aqueous solution containing 185 g of sodium potassium tartrate. Then, 5 g of crystalline phenol and 5 g of sodium sulfite were added, and the solution was stirred until dissolved. After cooling, distilled water was added to reach a volume of 1,000 ml, and the DNS reagent was stored in a brown bottle for future use. The glucose solution (0, 0.2, 0.4, 0.6, 0.8, 1.0, 1.2, 1.4, and 1.6 ml) was transferred into a 25‐mL stoppered colorimetric tube by pipette. Distilled water was then added into the tube to make the total volume 2 ml, after which 1.5 ml of the DNS reagent was added. Each tube was shaken, heated in a boiling water bath for 5 min, and then removed and cooled to room temperature with tap water. Distilled water was then added to a volume of 25 ml, and the sample was shaken to uniformity again. Zeroing was performed with a No. 0 tube as a blank control. The absorbance was measured at a wavelength of 510 nm. The standard curve was drawn taking the glucose content (in mg) as the ordinate and the absorbance as the abscissa.

#### 2.4.2 Analysis of reducing sugar (glucose)

As we know, polysaccharides in rice are mainly starches, and rice also contains a very small amount of dietary fibers, both starches and dietary fibers consist of glucoses, so the final hydrolysis products of rice by porcine pancreatic α‐amylase and glucoamylase (amyloglucosidase from aspergillus niger) are glucose, and there are no other reducing sugars present in the hydrolysis products. Therefore, the content of the reducing sugar determined by the DNS method is actually the content of glucose.

DNS method (Miller, 1959) was used to analyze the reducing sugar (glucose) content in the samples with some modifications. Briefly, the DNS reagent (1.5 ml) was added to 2 ml of sample solutions (sample solutions are diluted, if required, so that their concentrations of reducing sugar are within the range of 0.1 to 0.8 mg/ml) by dilution with distilled water, and the rest of the operation was the same as it is mentioned in section 2.4.1. According to the absorbance measured by the sample solution, the corresponding reducing sugar content was found from the standard curve. The reducing sugar (glucose) content in the sample was calculated as

$$\text{Reducing sugar}\;\left(\text{glucose}\right)\left(\;\text{\textbackslash{}\%}\;\right)=\frac{\text{Reducing sugar content obtained from the curve}\;\left(\text{mg}\right)\times \;\text{total volume of extract solution}}{\text{Volume taken for measurement}\;\times \;\text{sample weight}\;\left(\text{g}\right)\times 1000}\times 100$$

#### 2.4.3 RS, rapid digesting starch, and slow digesting starch analysis

The Englyst et al. (1992) method was applied with some modifications. Briefly, a sample (200 mg) was placed in a test tube. 15 ml of sodium acetate buffer (pH 5.2, 0.2 mol/l) was added and the solution was mixed, after which 10 ml of porcine pancreatic α‐amylase (290 U/ml) and glucoamylase (15 U/ml) were added. The mixture was placed in a water bath at constant temperature of 37°C and being shaken constantly. After hydrolysis for 20 min and 120 min, the glucose content was determined by colorimetric method at 510 nm. The slow digesting starch (SDS), rapid digesting starch (RDS), and RS contents were calculated as.

$$\begin{array}{c} \text{SDS}\%\;=\;\left({\text{G}}_{120}-{\text{G}}_{20}\right)\times 0\text{.9/W}\\ \text{RDS}\%\;=\;\left({\text{G}}_{20}-\text{FG}\right)\times 0\text{.9/W}\\ \text{RS}\%\;=\;\left(\text{W}-\text{SDS}-\text{RDS}\right)\text{/W} \end{array}$$

where G_20_ is the amount of glucose generated after hydrolysis of starch for 20 min (in mg), G_120_ is the amount of glucose generated after hydrolysis of starch for 120 min (in mg), FG is the free glucose content before enzymatic hydrolysis (in mg), and W is the total starch amount (in mg).

#### 2.4.4 Estimated glycemic index analysis

The estimated glycemic index (EGI) was determined by the Goñi, Garcia‐Alonso, and Saura‐Calixto (1997) and Englyst, Englyst, Hudson, Cole, and Cummings (1999) methods: Each sample solution of the raw material corresponding to 50 mg of starch was taken separately, and 10 ml of pH 1.5 HCl‐KCl buffer was added. The mixture was mixed with 0.2 ml of pepsin solution (0.1 g/ml) and shaken in a water bath at 40°C for 1 hr. The sodium acetate buffer (pH 6.9, 0.5 mol/L) was then added to achieve a volume of 25 ml, after which 5 ml of α‐amylase (2.6 IU) was added, and the mixture was shaken in a water bath at 37°C. At the following hydrolysis times, 0, 30, 60, 90, 120, 150, and 180 min, 1 ml of the digested sample solution was taken. The enzyme was deactivated in a water bath at 100°C for 5 min. After dilution to the appropriate concentration, the reducing sugar content in the digestive sample was measured by the DNS method using the diluted solution (2 ml).

$$\text{Percentage of starch hydrolysis}\;\left(\;\text{\textbackslash{}\%}\;\right)=\frac{\text{Glucose content in digestive sample at sampling time}\;\times 0.9}{\text{Total starch amount}}\times 100$$

The starch hydrolysis curve was drawn using the percentage of starch hydrolysis as the ordinate and the time as the abscissa. The area under the curve (AUC) was calculated. The starch hydrolysis index (HI) of the sample was obtained. The HI and EGI were calculated using the following equations:

$$\begin{array}{cc} \text{HI}\; & ={\text{AUC}}_{\text{sample}}/{\text{AUC}}_{\text{reference food (white bread)}}\times 100\\ \text{EGI}\; & =39.71+(0.549\times HI) \end{array}$$

#### 2.4.5 Observation via scanning electron microscope

The observation of rice flour was performed on a scanning electron microscope (SU‐70, Hitachi, Japan). The dried sample powder was fixed on the sample stage with conductive double‐sided tape, and gold was sprayed under vacuum conditions. Images were taken at a magnification of 1,000 times.

### 2.5 Data analysis

The data were expressed as the mean ± standard deviation (mean ± *SD*). The significance tests of the differences were performed using the Duncan test in a one‐way ANOVA with SPSS13.0 software. A *p*‐value of .05 was used as the significance threshold. Microsoft Excel 2010 was used to draw the statistical charts.

## 3 RESULTS AND DISCUSSIONS

### 3.1 Results of the single factor test

#### 3.1.1 Effect of water content on RS content

**Figure 1:**
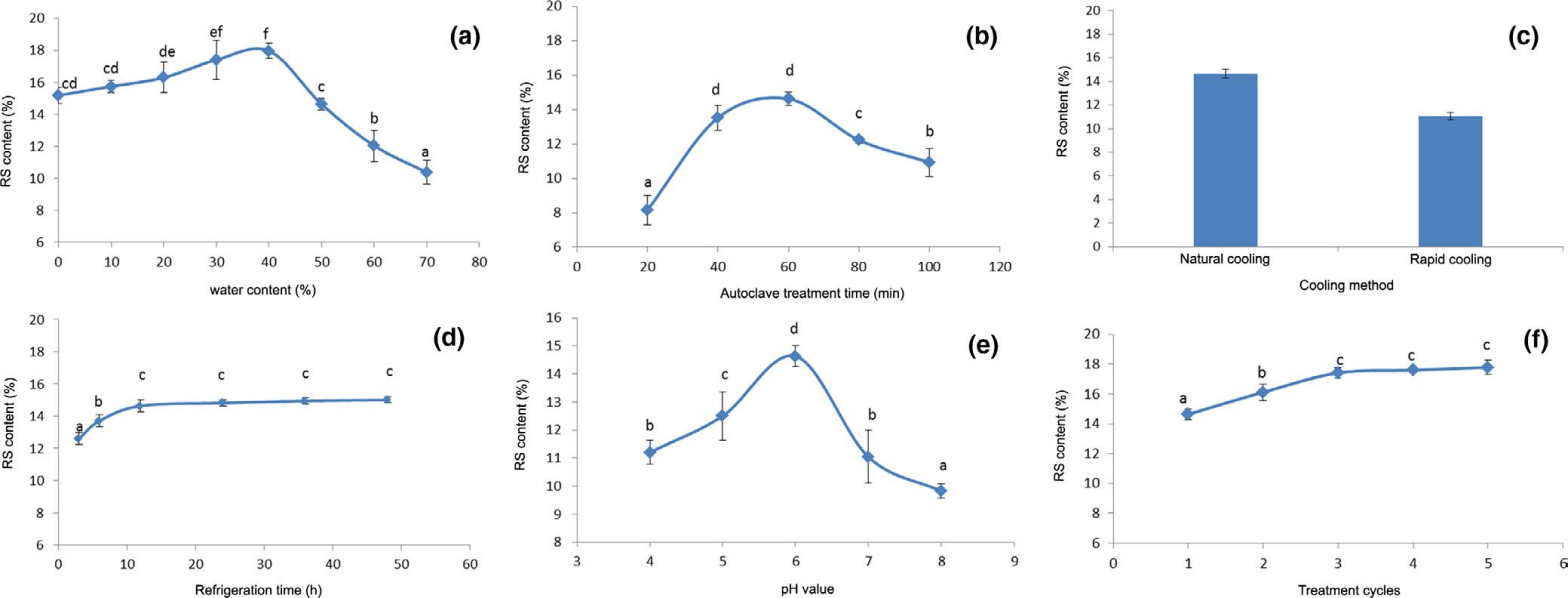
Effect of single factors on RS content

The effect of different water contents on RS content is shown in Figure 1a. It can be seen in Figure 1a that the RS content increased first and then decreased with an increasing amount of water. The RS content reached the maximum when the amount of water added was 40%. When the amount of water added is low, the starch could not be fully gelatinized, and the destruction of the starch molecular sequence is incomplete, resulting in insufficient amylose molecules for the formation of a double helix structure and inadequate aging, so the RS content will be relatively low (Ji et al., 2010; Li et al., 2010, 2015). However, if the amount of water added is too high, the distance between the starch molecules after starch gelatinization becomes too large, and then it is difficult for the amylose molecules to get close enough to form a double helix by intermolecular hydrogen bonding, ultimately resulting in a low RS content (Ji et al., 2010; Li et al., 2010, 2015). Therefore, the formation of RS requires a suitable level of water content.

This result is consistent with the result obtained by Li et al. (2010) with rice starch. The production of enzyme resistant starch involves retrogradation of starch molecules subsequent to gelatinization of the starches (Ashwar, Gani, Shah, Wani, & Masoodi, 2015; Thompson, 2000). In the autoclaving process, gelatinization of hydrated starch takes place during which amylose chains are leached out from the granules as random coils; in the cooling step, retrogradation of starch takes place during which the linear flexible amylose chains recrystallize and form tightly packed double helices stabilized by hydrogen bonds (Ashwar et al., 2015, 2016). The appearance of the amylose crystallization zone and tightly packed double helices could prevent the amylase from contacting the α‐1,4 glycosidic bond in the starch crystalline region and prevent the binding site of the amylase active center from binding to the starch molecule, thereby imparting the ability of amylose crystals to resist amylase digestion (Hoover & Zhou, 2003).

#### 3.1.2 Effect of autoclave treatment time on RS content

The effect of autoclave treatment time on RS content is shown in Figure 1b. It can be seen that the RS content increased first and then decreased with increasing autoclave treatment time, and at 60 min the RS content reached the highest value (14.72%). In the case of a shorter autoclave treatment time, the starch is not completely gelatinized, and the particles are not completely destroyed; thus, the amylose molecules will not be fully released and insufficient hydrolysis of amylose will occur as well (Onyango, Bley, Jacob, Henle, & Rohm, 2006; Yang et al., 2010). When the concentration of suitable substrate is too low and the reaction is insufficient, a low RS content will be resulted (Onyango et al., 2006). As the autoclave treatment time increases, the RS content gradually increases. However, if the autoclave treatment time is too long, undesirably high degree of starch hydrolysis would occur, resulting in the production of amylose with a relatively lower degree of polymerization; the longer the time, the more serious the hydrolysis and the lower the degree of starch polymerization (Yang et al., 2010). Short amylose molecules with low molecular weights will be generated by the hydrolysis of starch which occurs during autoclaving (Onyango et al., 2006). After starch gelatinization, the movement of short amylose molecules will be too fast, which is not conducive to the formation of RS (Ruan & Liu, 2006; Yang et al., 2010). The degree of polymerization of RS is approximately 50 to 60 (Goñi, García‐Diz, Mañas, & Saura‐Calixto, 1996), so starch chains that were too long or short are not suitable (Li et al., 2015; Liu et al., 2011; Pan et al., 2008; Ruan, Ma, Wu, Xiao, & Song, 2015; Yang et al., 2010). Therefore, the formation of RS requires a suitable autoclave treatment time. A time that was too long or short is not conducive to the formation of RS; these findings are consistent with the results obtained by other researches (Ji et al., 2010; Li et al., 2010, 2015).

#### 3.1.3 Effect of cooling method on RS content

The effect of different cooling methods on RS content is shown in Figure 1c. The amount of RS formed during rapid cooling was less than the amount formed during natural cooling. The formation of crystal nuclei occurs above the glass transition temperature (Zhao et al., 2013; Zhao et al., 2009), whereas crystallization is hindered below the glass transition temperature. The temperature was maintained during natural cooling, and the amylose molecules formed a double helix and became superimposed into crystal nuclei that grew into a larger crystalline region (Onyango et al., 2006). In case of rapid cooling, in which the sample was directly placed in a refrigerator at 4°C and temperature decreased rapidly, the amylose molecules did not have enough time to rearrange into a crystalline structure; thus, rapid cooling was not conducive to the completion of the reaction, resulting in a low RS content (Yang et al., 2010). Zhao et al. (2013) found that in the cooling process of starch slurry in “Yitang rice,” the RS formed from ice bath cooling was lower than the amount of RS formed from natural cooling by 12.3%. In this experiment, the mass fraction of RS formed from rapid cooling in the refrigerator at 4°C was 11.02%, while the amount formed from natural cooling was 14.63%. The RS content formed from rapid cooling was 3.61% lower than that formed from natural cooling, which was a significant difference, indicating that the cooling method had a significant effect on RS content.

#### 3.1.4 Effect of refrigeration time on RS content

The effect of refrigeration time on RS content is shown in Figure 1d. The RS content increased as the refrigeration time increased. The increase in RS content between 3 and 12 hr was particularly rapid, whereas after 24 hr, the increase of RS content was slow and no significant changes were observed. The aging of starch takes a relatively long time to complete (Onyango et al., 2006), and the experimental results showed that the aging of starch was faster in the early stage, and almost no starch continued aging when the aging process progressed to a certain extent. Our result was in agreement with those obtained by other investigators (Onyango et al., 2006; Pan et al., 2008). The reason for no significant increase in RS content after refrigeration time reaches to some certain levels may be due to the fact that the recrystallization of amylose mainly occurs in the early stage of starch retrogradation (Yang et al., 2010), and the temperature induced high viscosity limits further aging process (Onyango et al., 2006).

#### 3.1.5 Effect of pH on RS content

The effect of different pH values on RS content is shown in Figure 1e. It can be seen from the figure that with an increasing pH value, the RS content increased first and then decreased. The RS content reached a maximum value (14.8%) at a pH of 6. Suitable H^+^ concentration may promote the crystallization of starch molecules. Although starch could be well gelatinized under neutral conditions, the promoting effect is relatively low at low H^+^ concentrations. The intermolecular hydrogen bonding in starch is hindered in the case of high acidity or strong alkalinity, which are not conducive to the completion of the reaction. When the system is under slightly acidic conditions, the starch is moderately hydrolyzed, and the amount of short amylose increases, which is a raw material substrate suitable for the formation of RS. Li et al. (2009) prepared oat RS by the autoclave method, in which the highest content was observed at pH 6.2, Kang, Song, Liu, Cui, and Li (2019) observed that highest content of RS in autoclaved sweet potato flour occurred at a pH of 6.0, and Song et al. (2014) found that highest content of RS in *Dioscorea opposita* Thunb. was achieved when it was autoclaved at a pH range of 6.0 to 7.0; their findings are consistent with the results obtained in this study.

#### 3.1.6 Effect of the number of treatment cycles on RS content

The effect of the number of treatment cycles on RS content is shown in Figure 1f. The RS content increased with the number of treatment cycles; however, the RS content increased slowly after three cycles. Nie (2008) reported that potato starch content increased from 9.77% to 16.58% after five cycles of autoclaving. Tang, Liu, Huang, and Wang (2013) prepared wheat resistant starch by autoclave method and achieved relatively high RS content after six treatment cycles. In this experiment, the RS content increased from 14.63% to 17.77% after five cycles of autoclaving of intact rice grains, which indicated that the autoclave‐cooling process is beneficial to the ordering of starch molecules.

### 3.2 Selection of analytical factors

It was found in the single factor experiment that the results obtained from natural cooling were significantly better than those obtained from rapid cooling, and the increase of RS content was not significant after three cycles. Based on these results, cooling method was set to natural cooling, and the number of treatment cycles was set to three in later experiments, while the water content, autoclave treatment time, refrigeration time, and pH value were selected for further optimization, and the Box–Behnken in the Design‐Expert® software v8.0.6 was used to design the four‐factor three‐level response surface (Yang & Gao, 2005) optimization scheme. Factor codes for water content, pH, autoclave time, and refrigeration time were A, B, C, and D, respectively, and the levels for water content were set at 30, 40, and 50%, pH at 5, 6, and 7, autoclave time at 40, 60, and 80 min, and refrigeration time at 6, 12, and 18 hr, respectively.

### 3.3 Optimization of preparation conditions by the response surface method

#### 3.3.1 Model establishment and significance test

Response surface design and results are shown in Table 1. According to the data in Table 1, multiple regression fitting was performed using the Design‐Expert v8.0.6 software to obtain the quadratic multiple regression model equation showing the relationship between the predicted value of RS yield (Y) and the independent variables [water content (A), pH value (B), autoclave treatment time (C), and refrigerated time (D)]:

$$\text{RS}=17\text{.08}+1\text{.10A}-0\text{.77B - 0.10C}+0\text{.89D}+0\text{.50AB}+0\text{.87AC}-0\text{.50AD}-1\text{.50BC}+0\text{.50BD}+0\text{.000CD}-2{\text{.11A}}^{2}-3{\text{.37B}}^{2}-1{\text{.17C}}^{2}-0{\text{.46D}}^{2}$$

**Table 1:** Design and results of response surface test

Test number	A (water content, %)	B (pH value)	C (autoclave time, min)	D (refrigeration time, h)	Y (RS content, %)	Test number	A (water content, %)	B (pH value)	C (autoclave time, min)	D (refrigeration time, h)	Y (RS content, %)
1	0	1	−1	0	11.94	16	1	−1	0	0	12.36
2	1	0	0	1	16.14	17	0	0	0	0	16.7
3	−1	−1	0	0	11.59	18	0	−1	0	1	13.12
4	0	0	1	−1	13.18	19	−1	0	1	0	10.77
5	1	0	1	0	15.54	20	0	1	0	−1	12.13
6	1	0	0	−1	15.02	21	0	0	0	0	15.9
7	0	0	0	0	17.8	22	0	−1	1	0	16.52
8	0	−1	−1	0	11.81	23	0	0	0	0	18.5
9	−1	0	0	1	15.37	24	0	0	−1	1	17.59
10	0	0	0	0	16.5	25	0	1	1	0	10.65
11	0	0	−1	−1	14.47	26	0	0	1	1	16.3
12	0	1	0	1	13.25	27	1	1	0	0	12.49
13	1	0	−1	0	14.83	28	−1	0	−1	0	13.54
14	−1	0	0	−1	12.25	29	−1	1	0	0	9.72
15	0	−1	0	−1	14.00						

The obtained quadratic regression equation was analyzed by variance, and the results are shown in Table 2. The variance analysis of the model equation showed that the *F* value was 7.27 and the regression model was significant (*p* < .0003). The P‐value for the lack of fit was 0.4298 (greater than 0.05). The lack of fit was not significant, indicating that the test fitted well with the equation. The correlation coefficient, R^2^, was 0.866. There was a high correlation between the independent variables and factors. The test error was small, indicating that these results could be used for the analysis and prediction of the preparation process for rice with a high RS content. The first‐degree term of water content A (*p* = .0055) had a highly significant effect on the RS content. The pH value B (*p* = .0374) and refrigeration time D (*p* = .0182) also had significant effects on the RS content, while the autoclave treatment time C (*p* = .7655) did not. The interaction between autoclave treatment time and pH value was significant (*p* = .0213), while the interactions between the other factors were not significant. It can be seen from the *F* value that the order of the effect of each factor on RS content was as follows: water content (A)> refrigeration time (D)> pH value (B)> autoclave treatment time (C).

**Table 2:** Significance test of regression equation coefficient

Source of variation	Sum of square	Degree of freedom	Mean square	*F* value	*p*‐value	Significance
Model	136.4	14	9.74	7.27	.0003	**
A	14.39	1	14.39	10.73	.0055	**
B	7.08	1	7.08	5.28	.0374	*
C	0.12	1	0.12	0.093	.7655	
D	9.58	1	9.58	7.14	.0182	*
AB	1	1	1	0.75	.4023	
AC	3.03	1	3.03	2.26	.1551	
AD	1	1	1	0.75	.4023	
BC	9	1	9	6.71	.0213	*
BD	1	1	1	0.75	.4023	
CD	0	1	0	0	1	
A2	28.92	1	28.92	21.58	.0004	**
B2	73.52	1	73.52	54.84	<.0001	**
C2	8.9	1	8.9	6.64	.0219	*
D2	1.38	1	1.38	1.03	.3271	
Residual	18.77	14	1.34			
Lack of fit	14.36	10	1.44	1.3	.4298	
Pure error	4.41	4	1.1			
Total variation	155.17	28				

* Significant difference (*p* ≤ .05).

** Significant difference (*p* ≤ .01).

#### 3.3.2 Response surface interaction analysis

The response surface graph was a three‐dimensional map of the surface formed by the response value Y with respect to each test factor. In order to visualize the interaction between two factors, the contour map and response surface graph of the two factors were plotted.

**Figure 2:**
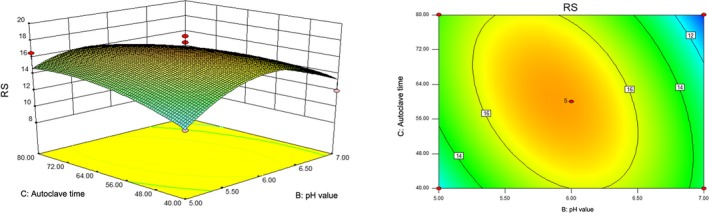
Response surface and contour map of autoclave time and pH value to RS content

An elliptical contour indicates a strong interaction between the two factors, and a circular contour indicates a weak interaction (Krishnaswamy, Orsat, Gariépy, & Thangavel, 2013). A relative steep slope of the response surface indicates that the influence of the processing condition on the response value is large, while a relatively smooth slope of the response surface indicates that the influence of the processing condition on the response value is small (Cao, 2014). Figure 2 shows that the opening of the response surface was downward. When the autoclave treatment time was constant, the RS content initially increased and then decreased with an increasing pH; in general, the trend was clear. When the pH value was constant, the RS content exhibited a trend of increasing first and then decreasing with the increase of autoclave treatment time, yet this trend was not significant. As shown in Figure 2, the contour plot was flat and appeared elliptical, indicating that the interaction between the autoclave treatment time and the pH value was significant. As each factor changed, the response value Y increased to the maximum value (Sun et al., 2017), which was the maximum value of the RS content, and the optimal process conditions were then obtained.

#### 3.3.3 Determination and verification of optimal preparation conditions

The optimal conditions for the model were obtained when the water content was 41.63%, pH was 5.95, autoclave treatment time was 61 min, and refrigeration time was 17.12 hr, as analyzed by the Design‐Expert v8.0.6 software. Under these conditions, the theoretical RS content was 17.57%. The test was then carried out under the optimal conditions. After three repeated tests, the average RS content was 17.11% with a small relative error (0.51). The actual value was close to the theoretical value, indicating that the optimization results were reliable and practical. Li et al. (2010) conducted a similar test using rice starch as the raw material with the autoclave method. A yield of 9.54% was obtained, yet other nutrients in the rice were wasted. Zhao et al. (2013) conducted a test using “Yitang rice”; a yield of 20.1% was obtained, which was higher than the RS content obtained in this study. The raw material used in Zhao *et al.*’s experiment was a rice variety with a higher natural RS content (8.17%) (Yang et al., 2006), and the RS content increased by 11.93% after treatment. However, the raw material used in our study was traditional *indica* rice. The RS content before treatment was only 1.9%, and the RS content increased by approximately 15% after treatment. In addition, the preparation method used in Zhao *et al.*’s experiment included pulverizing the rice into slurry. In contrast, intact rice grains were used in our study, which were easier to dry in the drying process, and the shape of the rice grains was maintained for the treated rice. For people accustomed to eating rice, the original grain shape of the rice is preferred over pulverized rice. Zhou et al. (2014) used the double‐enzymatic treatment for rice starch and obtained a higher RS content. However, since the raw material used was rice starch and its cost is high, this process would be difficult to apply in industrial production. Furthermore, this treatment could not be used for processing intact rice grains. In the present study, the process was optimized by adopting a different approach, and intact rice grains instead of rice starch or pulverized rice samples were used as the raw material to avoid the loss of nutrients while improving the RS content; these results are more suitable for industrial production.

### 3.4 Effect of autoclaving on the in vitro digestion characteristics of rice

The hydrolysis curve was drawn with the hydrolysis rate as the ordinate and the time as the abscissa, and the curve followed the first‐order reaction equation (the hydrolysis curve not shown here). The AUC was calculated to obtain the hydrolysis index (HI) and EGI values of the sample.

The results showed that as the hydrolysis reaction time increased, the hydrolysis of the uncooked autoclaved rice and the uncooked untreated rice both continuously increased, but the hydrolysis of the untreated rice was always less than that of the autoclaved rice. The reason that the hydrolysis of the untreated rice was lower than that of the autoclaved rice was that the untreated rice has not been subjected to autoclaving, so the structure of the starch remained unchanged, resulting in the amylase not being in full contact with the starch molecules. This result was different from that of Dioscorea Opposita Thunb. obtained by Song et al. (2014), which untreated crude starch of Dioscorea Opposita Thunb. was more readily digested than treated starch with higher RS contents. The reason for this may be due to the fact that the rice samples in our study are different in composition from the starch samples of Dioscorea Opposita Thunb., where the rice samples contain protein, vitamins, and minerals in addition to starch, while the starch samples of Dioscorea Opposita Thunb contain starch only, and the starch extraction process of Dioscorea Opposita Thunb may exert an impact on the structure of the starch samples.

The results also showed that the hydrolysis of the cooked autoclaved rice and the cooked untreated rice both increased. However, the hydrolysis of the autoclaved rice was much smaller than that of the untreated rice, which revealed results opposite to the results obtained from uncooked rice.

The cooking process before measurement caused gelatinization of both samples, which caused the amylase to react more readily with the starch in the rice samples. As a result, the hydrolysis of any rice sample subjected to cooking before measurement was higher than that of the same rice sample not subjected to cooking. However, the RS content of the autoclaved rice was higher than that of the untreated rice, and the effect of cooking before measurement on the hydrolysis for the autoclaved rice was relatively small, thus resulted in a much lower hydrolysis rate measured after cooking for the autoclaved rice than the untreated rice. It is well known that rice must be cooked in order to get better absorption. Therefore, the HI and EGI results obtained after cooking better reflect the absorption of rice in the human body.

Table 3 shows the HI and EGI of the autoclaved rice and the untreated rice measured with and without cooking. When measured without cooking, the HI and EGI of the autoclaved rice were higher than those of the untreated rice, while the HI and EGI of the autoclaved rice measured with cooking were lower than those of the untreated rice.

**Table 3:** HI and EGI of uncooked and cooked samples

Sample	Hydrolysis index (HI)		Estimated glycemic index (EGI)	
	Uncooked	Cooked	Uncooked	Cooked
Untreated rice	16.84 ± 0.27^a,A^	70.37 ± 0.46^b,B^	48.95 ± 0.15 ^a,A^	78.35 ± 0.25 ^b,B^
Autoclaved rice	34.03 ± 0.06^b,A^	48.03 ± 0.17^a,B^	58.39 ± 0.03 ^b,A^	66.08 ± 0.09 ^a,B^

Note

Different lowercase letters in the same column indicate that the values of that column had a significant difference. Different capital letters in the same row within the same index indicate that the values of that row had a significant difference.

Food with GI values less than 55, between 55 and 75, and above 75 is called low‐GI food, medium‐GI food, and high‐GI food, respectively. For people with diabetes, low‐GI foods should be consumed in order to maintain blood sugar stability after meals and ensure that blood sugar content does not rise sharply and adversely affect the individual's health. However, in regard to the eating habits of people in China and many other parts of the world, rice is considered a traditional staple food. The results in this study showed that the HI and EGI values of rice after cooking reached 70.37 and 78.35, respectively, indicating that rice is high‐GI food. After autoclaving, the HI of the rice measured with cooking decreased from 70.37 to 48.03, and the EGI values decreased from 78.35 to 66.08, indicating that the RS generated by autoclaving could significantly reduce the GI value of the rice.

### 3.5 Observation of rice flour samples using a scanning electron microscope

**Figure 3:**
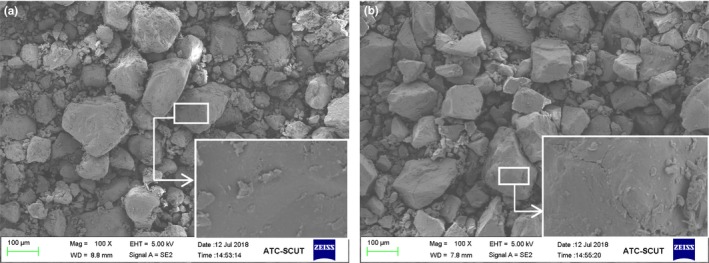
Scanning electron microscope images: (a) untreated rice; (b) autoclaved rice

Figure 3 shows that the morphologies of granules between the autoclaved rice flour and the untreated rice flour were similar. However, the surface of the granules of autoclaved rice flour had some long cracks and a small amount of debris, which was due to the fact that during the repeated autoclave treatments, the amylose molecules were dissolved and recrystallized, which resulted in cracks in the rice flour particles as well as a rough appearance. Wang, Liu, Zhang, and Huang (2012) used the autoclave method to prepare wheat RS. It was discovered by electron micrographs that the granules of wheat RS were irregular, the surface was not smooth, and that there were folds and gullies; these findings are similar to the results obtained in our study.

## 4 CONCLUSIONS

In this study, rice grain was treated using the autoclave method. The single factor test showed that a water content of 30%–50%, a pH value of 5–7, an autoclave treatment time of 40–80 min, and a refrigeration time of 6–18 hr produced relatively high RS contents, which provided a basis for the design of the response surface test. Through the optimization of the response surface test, optimal conditions were obtained when the water content was 41.63%, the pH value was 5.95, the autoclave treatment time was 61 min, and the refrigeration time was 17.12 hr. Under these conditions, the RS content was 17.57%. Autoclaving greatly decreased the HI and EGI of the rice measured with cooking from 70.37 to 48.03, and from 78.35 to 66.08, respectively. Thus far, there has been no report on improving the rice RS content through the treatment of intact rice grains. The concept adopted in this study is different from other ideas of using starch as the raw material to increase RS content. The raw material used in this study is intact rice grains, which avoids the difficulty of drying the starch slurry in the later stages. In addition, the protein, fat, vitamins, and minerals were comprehensively utilized in the prepared rice samples.

Numerous studies have shown that fat and protein influence the digestibility of starch. At present, the EGI of rice obtained in this study is still high for diabetics. The autoclaved rice can be combined with fat and protein to further reduce the digestibility of starch in rice. Research on further reducing GI values of rice is underway in our laboratory with the aim of providing new research concepts and specialized ingredients for the development of diabetes‐specific foods.

## CONFLICT OF INTEREST

The authors declare that they do not have any conflict of interest.

## INFORMED CONSENT

Written informed consent was obtained from all study participants.

## ETHICAL REVIEW

This study does not involve any human or animal testing.
